# Efficacy of *Nigella sativa* L. and *Trigonella foenum-*graecum straw ethanolic extracts against some tomato pests in a greenhouse

**DOI:** 10.1038/s41598-025-26428-9

**Published:** 2025-11-21

**Authors:** Hanaa E. Sadek, Waleed Abouamer, Huda H. Elbehery, Tarek Abd El-wahab, Hany Hussein, Amr Farouk

**Affiliations:** 1 Pests and Plant Protection Department, Institute of Agricultural and Biological Research, National Research Centre, Cairo, 12622 Egypt; 2 Plant Protection Department, Faculty of Agriculture, Al-Azhar University, Cairo, 11651 Egypt; 3 Flavor and Aroma Chemistry Department, National Research Centre, Cairo, 12622 Egypt

**Keywords:** Tomato crop, *Nigella sativa* L., *Trigonella foenum-*graecum, Postharvest straw, Insecticidal activity, Greenhouse, In-silico, Ecology, Plant sciences

## Abstract

The tomato (*Lycopersicon esculentum*) is a crucial vegetable crop worldwide, but various pests threaten its yield. Excess food and agricultural waste create health and environmental issues. This study evaluated the pesticidal activity of ethanolic extracts from *Nigella sativa* and *Trigonella foenum-graecum* straw ethanolic extracts against *Amrasca biguttula biguttula*, *Liriomyza trifolii*, and *Tuta absoluta* under greenhouse conditions. Tomato leaflets were collected from treated plots before spraying and examined in the lab for pests. Then, infestation rates were assessed 7 days after spraying by comparing the number of larvae to that of an untreated control. All treatments showed significant differences in the mean number of recorded pest infestations after the first and second sprays compared to the control. The 5% *T. foenum-graecum* extract was the most effective, reducing pests by 78.98%, 81.94%, and 28.03%, respectively, while *N. sativa* extract caused an 88.75% reduction in *A. biguttula*. The high-performance liquid chromatography analysis identified 18 phenolic compounds in *N. sativa* straw extract, with the predominance of catechol (330.14 µg/mL), chlorogenic acid (169.23 µg/mL), catechin (94.07 µg/mL), naringenin (91.99 µg/mL), and rutin (78.16 µg/mL). A similar profile was observed for the ethanolic extract of *T. foenum-graecum* straw, with some quantitative differences, where ellagic acid (287.13 µg/mL), gallic acid (188.89 µg/mL), naringenin (48.71 µg/mL), rutin (34.99 µg/mL), and catechin (33.97 µg/mL) were the major phenolics in the extract. In line with the above findings, rutin, chlorogenic acid, and daidzein showed the highest in-silico docking scores against AChE, GABA_(A)_, and RyR enzymes compared to the controls. These results suggest that agricultural waste from *N. sativa* and *T. foenum-graecum* can serve as novel, environmentally friendly bioinsecticides.

## Introduction

The tomato, *Lycopersicon esculentum* Mill, is a significant vegetative crop farmed worldwide in various climates, either as an export crop or for domestic consumption^1^. Tomatoes can be cultivated in greenhouses or open fields throughout various climate zones^2^. Given their abundance of essential minerals, vitamins, amino acids, carbohydrates, β-carotene, flavonoids, vitamin C, and hydroxycinnamic acid derivatives, tomatoes are considered protective foods. Furthermore, the tomato crop has become increasingly important in light of the new findings about lycopene’s antioxidant and anticancer properties^3^. According to the FAO^4^, *Solanum lycopersicum* (tomato) is considered the second most important fruit worldwide.

Pests are among the many biotic factors contributing to the decline in tomato yield. Aphids, spider mites, leafhoppers, thrips, and whiteflies are among the worldwide plant pests that have severely reduced crop yields, primarily in the *Cucurbitaceae*,* Fabaceae*, and Solanaceae families^5^. Tomatoes are one of the most produced vegetables globally, with 192 million tons in 2023. Up to 40% of global crop production is lost annually due to pests and diseases, resulting in economic losses of over USD 220 billion, including USD 70 billion from invasive insects^6^. In Egypt, the fifth-largest tomato producer, tomatoes are a vital crop, occupying 469,000 feddans in 2015, or 32% of the total vegetable cultivation area^7^. In Egypt, the tomato crop is threatened by an invasive pest, the tomato leaf miner or *Tuta absoluta* (Meyrick) (Lepidoptera: Gelechiidae), which is considered one of the most important lepidopterous pests associated with tomatoes, *L. esculentum*^8^. The larvae of *T. absoluta* feed on the leaves, stems, and fruits of tomatoes, severely reducing productivity in both greenhouse and open-field tomato crops. Serpentine leaf miner *Liriomyza trifolii* Burgess (Diptera: Agromyzidae) and Leafhopper *Amrasca biguttula biguttula* are the most common pests attacking the tomato crop. *L. trifolii* is a significant insect pest of vegetables, particularly tomato crops in Mediterranean countries and worldwide^9^. Bruce et al.^10^ reported that greenhouse vegetable growers in 14 of Egypt’s 26 governorates were examined between May and July 2016. *T. absoluta* (Meyrick) (Lepidoptera: Gelechiidae) (40.7%) and nematodes (39%) were relatively abundant throughout locations. Other arthropod pests mentioned include the jassid bug, *A. biguttula* (Ishida) (Hemiptera: Cicadellidae), and leaf miners.

Traditional pesticides are often used to manage pests on tomatoes. However, due to their widespread application in agriculture, insect populations are becoming increasingly resistant to them, and there are also possible concerns and hazards for the environment and non-target organisms^11^. Therefore, it is urgently necessary to develop more environmentally friendly insecticides as part of more durable Integrated Pest Management (IPM) programs against pests due to the drawbacks of commercial insecticide use^12^. The botanical extracts serve as a sustainable alternative to synthetic pesticides, as they possess bio-insecticide properties and a minimal negative environmental impact^13^. Applying botanical extracts as part of an integrated pest management (IPM) program is considered an eco-friendly choice for pest control.

The yearly global production of *Nigella sativa* L. or black cumin oil is approximately 600,000 tons^14^, with India holding an 86% share, followed by Iran (4%), Syria (3%), and Turkey (2%). The remaining 5% is sourced from countries like Egypt, Pakistan, and Afghanistan. Egypt is also a notable producer of *Trigonella foenum-graecum* L., also known as fenugreek, with an estimated 25,000 metric tons expected in 2025, primarily from regions such as Fayoum, Minya, and Beni Suef^15^. *N. sativa* L., found in regions like the Middle East and North Africa, offers various therapeutic benefits, including antiseptic, anti-inflammatory, and antioxidant properties. Research shows it significantly inhibits acetylcholinesterase in *Agrotis ipsilon*^16^. *T. foenum-graecum* L. is an aromatic plant from the Fabaceae family used as a spice and herb. Its leaf extract exhibits pesticidal activity against *Rhyzopertha dominica* and *T. absoluta*^17]– [18^.

Agricultural and food-industrial processes generate significant waste, including stalks, leaves, straw, and husks, which can pose health and environmental risks. Vast amounts of agricultural waste are accumulated, posing a global health and environmental concern, and may include bioactive phenolics. Therefore, food sustainability policy began to target these wastes as raw materials for the bioeconomy^19^. Specifically, in the case of *N. sativa* and *T. foenum-graecum*, the straw, the most notable post-harvest waste, consists of various dried plant parts. Farouk et al.^16^ have reported the phenolic content in *N. sativa* straw extract as a bioinsecticide in laboratory conditions against the *Agrotis ipsilon*. On the other hand, no information was reported regarding the bioactive constituents of *T. foenum-graecum* straw extract or its use as an environmentally friendly insecticide. As a novel perspective, the extraction of bioactive compounds from these agricultural wastes for use in crop protection as bioinsecticides presents a potential solution to the environmental and health gaps caused by waste generation and the use of synthetic pesticides. The widespread use of synthetic pesticides has led to problems such as pest resistance and contamination of vital resources, including water, air, and soil. To enhance crop production and address food crises sustainably while protecting consumer health, plant-derived pesticides offer a greener alternative. They are cost-effective, biodegradable, and eco-friendly, acting in a targeted way that reduces risks to humans and the environment^20^. Consequently, the current study aimed to investigate the insecticidal inhibitory potential of *N. sativa* and *T. foenum-graecum* straw ethanolic extracts against pests attacking *Lycopersicon esculentum* Mill (tomato) plants in greenhouse conditions. Screening for phenolics in both extracts was compared using High-Performance Liquid Chromatography. The mechanisms of interaction of the main phenolics present in both extracts were examined using a molecular docking assay against acetylcholinesterase (AChE), the γ-aminobutyric acid (GABA) receptor, and the ryanodine receptor (RyR), which are extensively used to evaluate the in silico insecticidal potential of synthetic and natural compounds^21^. The study suggests that utilizing postharvest agro-waste as a bioinsecticide can help manage agricultural waste while minimizing negative impacts on health and the environment.

## Materials and methods

### Agro-wastes and chemicals

*N. sativa* L. and *T. foenum-graecum* leaf and stem residues were collected in the last week of April 2022 from Dairut, Assiut, Upper Egypt, located at 27°11′N, 31°10′E, about 375 km from Cairo. The soil type in Dairut is clay, and the farm’s environmental conditions were characterized by an average temperature of 30 ± 2 °C and a relative humidity range of 15–20%. The seeds were directed to the food and pharmaceutical industries. A taxonomist at the National Research Center in Cairo identified the agro-wastes. Sigma-Aldrich (St. Louis, MO, USA) provided ethanol and phenolic standards; additional chemicals were purchased from Merck (Darmstadt, Germany).

### Ethanolic extract Preparation

*N. sativa* L. and *T. foenum-graecum* straw were dried and suspended in 70% ethanol (7:3 ethanol to water), which proved to be effective in phenolics extraction^22^, at a solid-to-solvent ratio of 10 g of the straw to 100 ml of the solvent (1:10 w/v). The mixture was stirred for 24 h, filtered through Whatman No. 1 filter paper, and the filtrate was vacuum-dried for further analysis^16^.

### High-Performance liquid chromatography (HPLC) analysis of the extracts

The *N. sativa* and *T. foenum-graecum* straw ethanolic extracts were analyzed for phenolic profiles using HPLC with an Agilent 1260 Infinity Quaternary LC System (Agilent, Waldbronn, Germany). Separation was achieved on an Agilent ZORBAX Eclipse Plus C18 column (4.6 mm × 250 mm, 5 μm) with a mobile phase of water (A) and 0.05% trifluoroacetic acid in acetonitrile (B) at a 0.9 ml/min flow rate. The mobile phase was programmed consecutively in a linear gradient as follows: 0 min (82% A); 0–5 min (80% A); 5–8 min (60% A); 8–12 min (60% A); 12–15 min (82% A); 15–16 min (82% A) and 16–20 (82%A). The multi-wavelength detector was set at 280 nm, with a 5 µl sample injected at 40 °C. The solvent was degassed in an ultrasonic bath before use. Peaks were compared to standards based on retention times and UV spectra, with detection limits ranging from 0.1 to 0.5 ppm, and a correlation coefficient (R²) of greater than 0.9993 for concentrations ranging from 0.5 to 200 ppm. Analysis performed in duplicate^23^.

### Field experiment

Field studies were conducted during the 2023 tomato growing season in the greenhouse at Bheira Governorate from mid-February 2023 to the end of May 2023. The experimental area was located at the agricultural experiment station of the National Research Center in the Nubaria region, Egypt (latitude 30.8667 °N, longitude 31.1667 °E, and a mean altitude of 21 m above sea level), approximately 140 km from Cairo. Tomato plants, *Lycopersicon esculentum* (Mill.) variety ‘ceren F1’ were planted in the greenhouse, which was divided into longitudinal plots using the Randomized Complete Block Design (RCBD). Three replicates were suggested for each planted tomato plot, which served as a treatment. The plants chosen as controls were untreated; they were just sprayed with water. The total number of plots was 9 (2.5 m in width × 3 m in length for each plot) with a 0.5 m spacing between plots. Each plot is divided into three replicates (10 plants for each). The plants were individually supported with a plastic rope. A buffer zone (50 cm) was maintained between the control (2.5 m) and treated plots (2.5 m) to minimize the possibility of contamination or spray drift interference. The application of two distinct insecticidal treatments was evaluated, with each treatment consisting of two consecutive sprays, separated by four weeks, along with a control treatment. The treatments included three different concentrations of the plant extracts (5%, 2.5%, and 1.25%), which were selected based on a preliminary experiment and a previously published study^16^. The efficiency of the tested extracts was estimated weekly by counting the target live pests, *Amrasca biguttula biguttula*,,*Liriomyza trifolii*, and *Tuta absoluta* larvae, on the lower surface of 30 tomato leaflets, in addition to untreated plants (Table 1).

### Sampling technique

Tomato leaflets were randomly collected from each replicate before spraying. The tomato plants (30 plants) were treated with each concentration. Ten leaves were collected from each plant in each treated plot. These samples were carefully placed in sample preservation bags and then transferred to the laboratory for examination of the different living stages that had been presented on the plant’s surface. The samples were stored in the refrigerator at 10 °C until all samples had been investigatedThe total numbers of each pest were recorded, and the mean numbers were calculated for the various concentrations of every pest.

By observing the symptoms of morphological alterations in the specimens obtained from the field treatments that were applied, the existence of *A. biguttula*,* L. trifolii*, and *T. absoluta* was verified. The infestation rate was recorded 7 days after each spray, considering the mean number of larvae/5 sampled plants/ replicate in each treatment compared with the untreated (control). The percentages of infestation reduction were calculated according to the method of^24^.

$${{\text{Reduction\% }}=\left[ {{\text{1}} - \left( {\frac{A}{B}X\frac{C}{D}} \right)} \right]X{\text{100}}}$$

Where A: The number of larvae after spray treatments; B: Number of larvae before the spray treatment; C: Number of larvae in the control before spray treatments; and D: Number of larvae in the control after spray treatments.

**Table 1:** A diagram illustrates the distribution of plant treatments with different concentrations of the extract in the greenhouse.

	Entrance							
	LINE 1			LINE 2			LINE 3	
A1	B1	C1	A2	B2	C2	A3	B3	C3
B1	C1	A1	B2	C2	A2	B3	C3	A3
C1	A1	B1	C2	A2	B2	C3	A3	B3
			Buffer		zone			
Control	Control	Control	Control	Control	Control	Control	Control	Control

Concentration A: 5%.

Concentration B: 2.5%.

Concentration C: 1.25%.

### Molecular Docking study

Enzyme crystal structures were downloaded from the Protein Data Bank (PDB) (https://www.rcsb.org/) on May 14, 2025, including human acetylcholinesterase (PDB ID: 4EY7), human gamma-aminobutyric acid receptor (PDB ID: 4COF), and rabbit ryanodine receptor 1 (PDB ID: 5C30). Water and ligand molecules were removed and protonated using PyMOL (version 2.5.1). The cocrystallized ligands were docked into the active pocket of each enzyme to validate the docking protocol, yielding an RMSD value of less than 2.0 Å using AutoDock 1.5.6 and Vina^25^. RMSD values were 0.757 (4EY7), 0.7308 (4COF), and 1.22 Å (5C30). Major phytochemicals as ligands were obtained from PubChem via http://pubchem.ncbi.nlm.nih.gov/ on January 27, 2023, and May 18, 2025, then optimized with Avogadro (version 1.2.0). The binding potential of the enzyme’s pockets was predicted using CB-DOCK2 and Discovery Studio. Based on the identified pocket residues and dimensions, docking was performed using AutoDock Vina through CB-DOCK2 (http://clab.labshare.cn/cb-dock/php/) on May 15, 2025^26^. The docked complexes were analyzed and visualized with Discovery Studio ver 25.1.0.24284^27^.

### Statistical analysis

Data were analyzed using one-way analysis of variance (ANOVA) using the SPSS version 26.0 computer program; means were compared using Duncan’s Multiple Range Test (*P* < 0.05).

## Results and discussion

### Phenolic compounds of the Agro-wastes using HPLC

In our study of the insecticidal activity of *N. sativa* straw extract against *Agrotis ipsilon*, we conducted a screening of phenolics in the ethanolic straw extract for the first time, as detailed in Table 2 (16). The HPLC analysis identified 18 phenolic compounds, including 3 simple phenols, 8 phenolic acids, and 7 flavonoids. The predominant phenolic compounds were catechol (330.14 µg/mL), chlorogenic acid (169.23 µg/mL), and gallic acid (110.93 µg/mL). The major flavonoids included catechin (94.07 µg/mL), naringenin (91.99 µg/mL), and rutin (78.16 µg/mL). According to Alu’datt et al.^28^, the above compounds were reported as major phenolic constituents of *N. sativa* seeds, with quantitative variations due to differences in geographic, climatic, and environmental conditions, as well as the part of the plant subjected to extraction and the type of solvent used.

A similar profile was observed for the ethanolic extract of *T. foenum-graecum* straw, although with some quantitative differences. The major phenolic acid constituents were ellagic acid (287.13 µg/mL), gallic acid (188.89 µg/mL), and chlorogenic acid (121.15 µg/mL). The predominant flavonoids in *T. foenum-graecum* straw were naringenin (48.71 µg/mL), rutin (34.99 µg/mL), and catechin (33.97 µg/mL). The *T. foenum-graecum* straw extract showed higher gallic, ellagic, and cinnamic acid concentrations than the *N. sativa* straw extract. It also contained larger amounts of apigenin, quercetin, and kaempferol. However, the *N. sativa* straw extract was superior in the remaining identified phenolics (Table 2).

Research indicates a lack of literature on the bioactive components of *T. foenum-graecum* straw extracts. However, the literature revealed common phenolics found in fenugreek seed extracts, which differ from the findings of the present study. Naidu et al.^29^ noted that fenugreek seed husks contain higher total polyphenols than their endosperms. Madany & Khalil^30^ identified several acids, including vanillic, syringic, and caffeic, in ethanolic extracts of fenugreek seeds. Benziane et al.^31^ found kaempferol, genistein, and vanillin in the aqueous maceration extract of Algerian seeds, while rutin and kaempferol were present in the decoction. Similarly, Hachouf et al.^32^ identified rutin and ferulic acid as the major components of the hydro-methanolic extract of Algerian seeds. According to Oufquir et al.^33^, HPLC analysis revealed the presence of rutin, caffeic, and syringic acids in both ungerminated and germinated fenugreek seeds, with concentrations increasing in the germinated seeds, particularly for syringic acid.

The results of investigations on phenolic compounds from medicinal or aromatic plants vary based on sample preparation, extraction methods, and environmental factors such as temperature, rainfall, and soil composition. Variables in the extraction process, such as sample-to-solvent ratios, solvent purity, temperature, time, number of extractions, and pH, also impact yield. Typically, solvents with varying polarity are used to extract these chemicals. Lohvina et al.^22^ found that the total phenolic content in fenugreek seeds decreased in the order of 70% ethanol > 96% ethanol > 50% ethanol > 30% ethanol, while the extraction yield was highest for 30% ethanol and lowest for 96% ethanol.

**Table 2:** Phenolic contents of *N. sativa* and *T. foenum-graecum* straw ethanolic extracts.

S/*N*	Compound	Quantities (µg/mL) *	
		*N*. sativa**	T. foenum-graecum
1	Gallic acid	110.93 ± 1.18	188.89 ± 1.33
2	Chlorogenic acid	169.23 ± 2.05	121.15 ± 1.07
3	Methyl gallate	25.43 ± 0.78	15.77 ± 0.43
4	Caffeic acid	18.21 ± 0.64	2.63 ± 0.06
5	Syringic acid	12.76 ± 0.44	9.74 ± 0.11
6	Catechol	330.14 ± 2.74	46.87 ± 0.77
7	Ellagic acid	25.54 ± 0.33	287.13 ± 2.11
8	Coumaric acid	33.64 ± 0.46	1.92 ± 0.04
9	Vanillin	43.55 ± 0.63	7.10 ± 0.33
10	Ferulic acid	20.17 ± 0.19	14.15 ± 0.48
11	Cinnamic acid	0.31 ± 0.08	1.35 ± 0.05
12	Catechin	94.07 ± 1.17	33.97 ± 0.39
13	Rutin	78.16 ± 1.37	34.99 ± 0.62
14	Naringenin	91.99 ± 1.15	48.71 ± 0.69
15	Daidzein	5.68 ± 0.15	1.08 ± 0.02
16	Quercetin	7.56 ± 0.74	9.85 ± 0.03
17	Apigenin	1.93 ± 0.14	4.31 ± 0.08
18	Kaempferol	-	7.92 ± 0.09
19	Hesperetin	8.48 ± 0.30	-

*Values represent averages ± standard deviations for triplicate experiments. **^16^.

### Insecticidal effect of straw extracts against some tomato pests

The results indicate that all concentrations of the *N. sativa* straw extract significantly reduced populations of the three major pests (*A. biguttula biguttula*, *L. trifolii*, and *T. absoluta*) compared to the untreated control. The 5% concentration showed the most significant suppression, followed by 2.5% and 1.25%. No significant differences were noted before spraying; however, after the first and second sprays, significant differences emerged (*p* < 0.05) from two weeks onward, with the 5% concentration being the most effective. The significant reduction in pest populations after one week of the second application compared with the control was also represented by statistical tests (F = 36.82; *df* = 3,8; *P* = 0.00; F = 18.04; *df* = 3,8; *P* = 0.00; and F = 6.87; *df* = 3,8; *P* = 0.01, respectively) as shown in Table 3. The F-value reflects the ratio of variance between treatment groups to the variance within groups. Higher F values indicate a stronger treatment effect relative to random variation. After eight weeks, pest populations in treated plots significantly declined to (1.67 ± 0.88, 4.33 ± 0.33, and 1.33 ± 0.67) compared to the control (10.67 ± 0.88, 21.67 ± 0.88, and 7.33 ± 1.33, respectively).

A similar trend was observed for *T. foenum-graecum* straw extract, where no significant variations in pest population density were observed between treatments before spraying (F = 5.62; *df* = 3,8; *P* = 0.03, F = 3.30; *df* = 3,8; *P* = 0.08, and F = 0.10; *df* = 3,8; *P* = 0.96). However, reductions in treated plots began one week after the spray and became more pronounced over time. By three weeks, pest populations of *A. biguttula biguttula*, *L. trifolii*, and *T. absoluta* treated with a 5% concentration were significantly reduced (0.67 ± 0.33, 10.33 ± 0.33, and 1.67 ± 0.33) compared to the untreated control (9.67, 22.33, and 8.33). This trend continued, with the lowest pest numbers consistently recorded in the 5% treatment (0.67 ± 0.67, 2.33 ± 0.33, and 1.67 ± 0.67) over the eight weeks (Table 4).

The findings indicate that all tested concentrations of botanical extracts significantly reduced populations of three main pests compared to the untreated control. The highest concentration (5%) showed the most notable suppression, followed by 2.5% and 1.25%. *A. biguttula biguttula* experienced a rapid decline within a week, while *L. trifolii* populations decreased gradually, showing prolonged suppression with higher doses. *T. absoluta* infestations also reduced in treated plots at later times with 2.5% and 5% treatments.

**Fig. 1:**
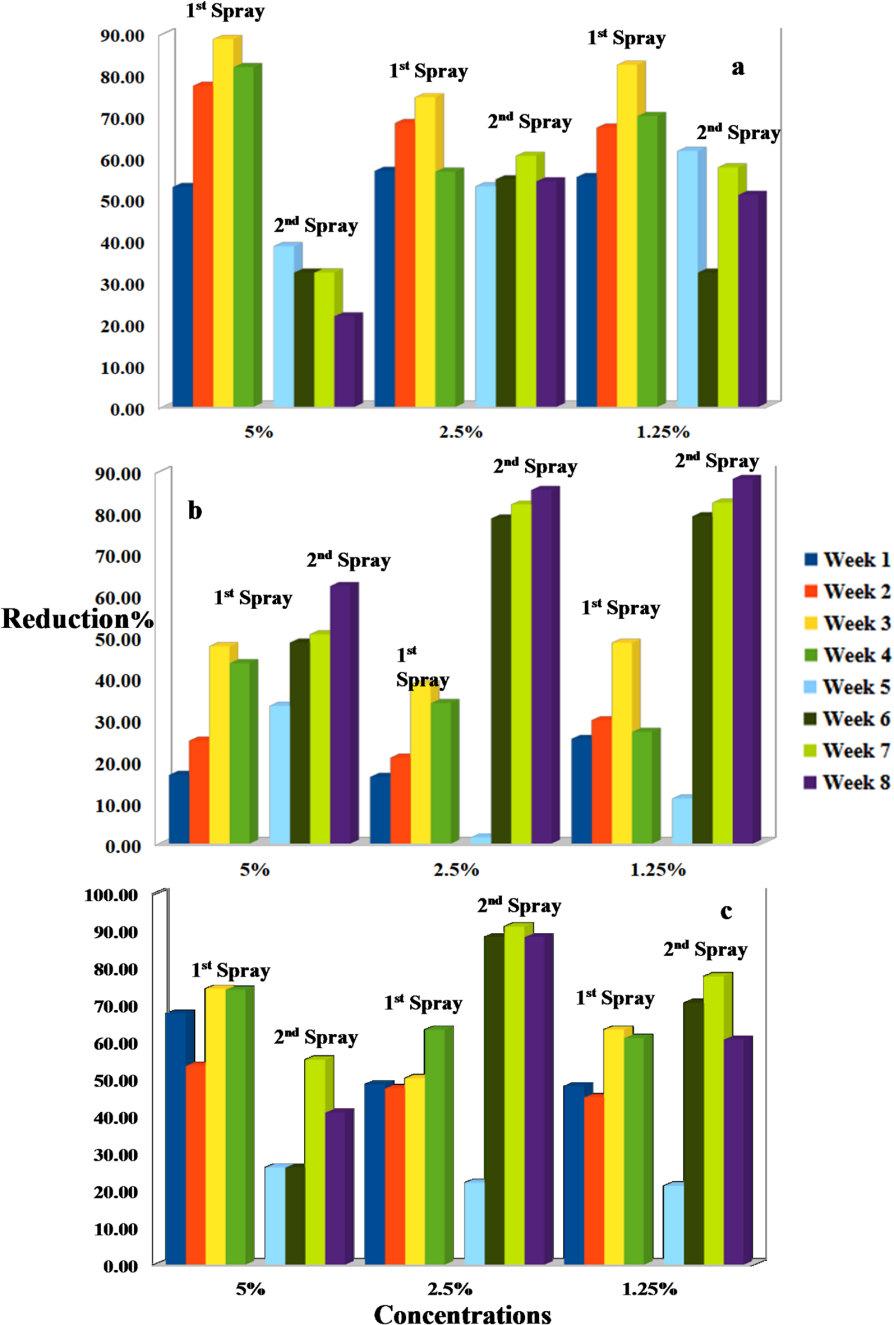
Reduction percentages of *Amrasca biguttula biguttula* (a), *Liriomyza trifolii Burgess* (b), and *Tuta absoluta* (c) after different time intervals of *N. sativa* extract treatments (first and second spray).

**Fig. 2:**
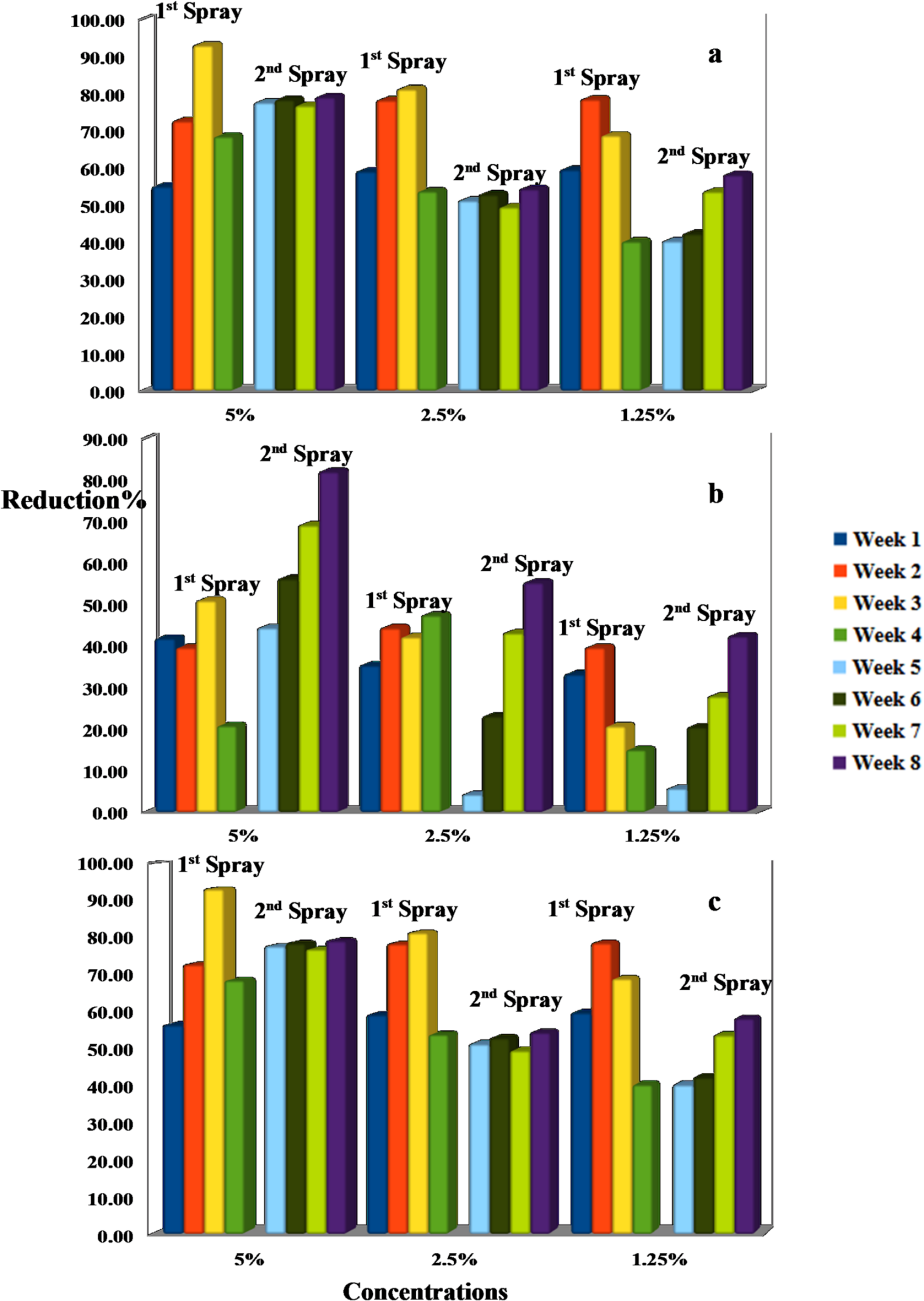
Reduction percentages of *Amrasca biguttula biguttula* (**a**), *Liriomyza trifolii Burgess* (**b**), and *Tuta absoluta* (**c**) after different time intervals of *T. foenum-graecum* extract treatments.

**Fig. 3:**
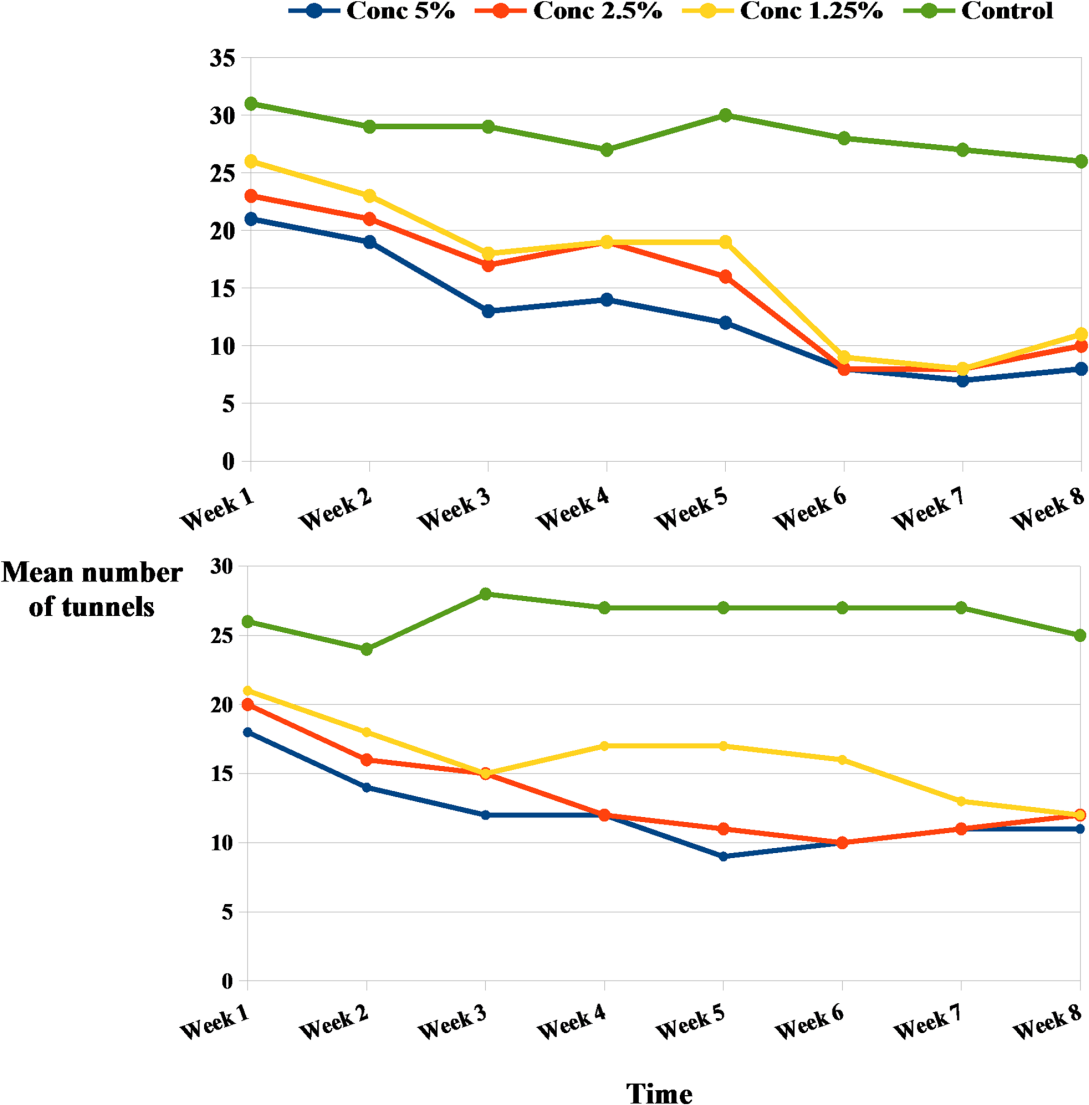
Mean number of tunnels recorded of *Liriomyza trifolii Burgess* after *N. sativa* extract (a) and *T. foenum-graecum* extract (b) treatments.

Meanwhile, the reduction percentages of *A. biguttula biguttula*,* Li. trifolii Burgess*, and *T. absoluta* infestation on the tomato plant after two different spray time intervals with *N. sativa* and *T. foenum-graecum* straw extracts are shown in Figs. 1 and 2. Figure 1 illustrates that higher concentrations of *N. sativa* straw extract result in greater pest reduction. The 5% concentration achieved an over 80% reduction in *A. biguttula biguttula*, sustained throughout the eight-week study with two spray applications. Lower concentrations (2.5% and 1.25%) also significantly reduced populations, especially after the second spray. For *L. trifolii*, the 5% concentration resulted in over 90% reduction after the initial application, with lower doses benefiting from cumulative effects. *T. absoluta* was also susceptible, with the 5% concentration maintaining significant reduction for weeks; even the 1.25% concentration led to over 80% reduction by the study’s end. Similar results were observed for *T. foenum-graecum* straw extract, which demonstrated rapid effects and sustained efficacy over eight weeks (Fig. 2). The highest concentration of *N. sativa* and *T. foenum-graecum* straw extracts resulted in the most significant reduction in the mean number of tunnels of *L. trifolii Burgess*, with values continuously declining from week 1 to week 8. The 2.5% concentration also significantly reduced tunneling when compared to the control, though not as efficiently as the 5%. The 1.25% concentration showed moderate suppression of tunneling, but its effect fluctuated over the weeks, particularly between weeks 3 and 5. Tunnel numbers in the control group remained high, with only slight declines over time, ranging from ~ 25 to 30 tunnels (Fig. 3).

These results are consistent with those reported by Farouk et al.^16^, who found that an ethanolic *N. sativa* straw extract revealed the antifeedant effect against the black cutworm. Our results supported Osman^34^, who found that plant extracts (*Nigella sativa*) led to significant effects on treated *S. littoralis* larvae compared to untreated ones. Based on our results, *T. foenum-graecum* exhibited a significant adverse effect on the population of *A. biguttula biguttula*, *L. trifolii*, and *T. absoluta* infesting tomato plants (Table 4). These results agreed with those of Pemonge et al.^35^, who confirmed that fenugreek extract induced mortality in adults of *Acanthoscelides obtectus*, resulting in decreased adult emergence compared to the control. Likewise, extensive in vivo research revealed that *T. foenum-graecum* seed extracts are highly effective against the lesser grain borer (*Rhyzopertha dominica*), which is resistant to pesticides. As a new pesticide candidate, the extract’s active ingredient or ingredients show promise^17^. A similar effect was reported by Hassan et al.^36^, who noted that the ethanolic extract was the most effective among all the extracts used to control the cotton leafworm. In the same line, Mohamed et al.^37^ found that the ethanol extract of *T. foenum-graecum* was shown to have the highest efficacy on the mealybug *Maconellicoccus hirsutus.*

The provided statistical results from the study on *N. sativa* and *T. foenum-graecum* extracts have significant ecological relevance. The data demonstrates a clear and effective alternative to synthetic pesticides, pointing towards a more sustainable and ecologically balanced approach to pest management. The statistical results are not just a record of efficacy; they are a strong indicator of a shift towards a more ecologically intelligent form of agriculture^38^. The use of *N. sativa* and *T. foenum-graecum* extracts aligns with the principles of sustainable pest management by reducing pollution and chemical residues, conserving biodiversity and ecosystem services, lowering the risk of pest resistance, and utilizing renewable, on-farm resources. This approach supports healthier crop yields while fostering a more resilient and balanced agricultural ecosystem. A 100% reduction in pest population is rarely achievable or necessary when using botanical extracts; however, a significant and acceptable reduction typically falls between 70 and 90%, depending on the specific pest, plant, and context of use. The “significance” of the reduction is judged by whether it prevents significant economic damage or reduces the population to a manageable level, allowing other integrated pest management (IPM) strategies to be effective^39^.

The recent review of Pereira et al.^38^ on the non-target effects of botanical pesticides in soil biology reveals significant knowledge gaps that complicate assessments of their actual impact. Many studies lack diversity in their target organisms, focusing primarily on earthworms, and present contradictory findings, making it difficult to determine whether these pesticides stimulate or inhibit microbial growth and soil enzyme activity. The degradation of botanical pesticides in soil and their metabolites, such as phenolics and flavonoids, is not well understood, creating a knowledge gap since breakdown products may have different effects on plant pathogens and non-target organisms. For instance, Lamiaceae essential oil residues, neem seed extracts, and garlic or mint essential oils, when used via fumigation, were designated as nontoxic to the egg parasitoid *Trichogramma sp*^40,41^. Overall, there was no relationship between the negative effects of bioinsecticides and the studied natural enemy (predators or parasitoids) under laboratory conditions^42^. Enhancements in predictive models for biodegradation, combined with the development of synthetic biotechnology, offer opportunities to better understand the impact of botanical pesticides and their metabolites on soil biology and biochemistry^43^.

**Table 3:** Effect of the *N. sativa* treatments against *Amrasca biguttula biguttula*, *Liriomiza trifolii*, and *Tuta absoluta* infesting tomato plants (first and second spray).

1st spray						2nd spray				
Concentrations	Pre-spray	One week	Two weeks	Three weeks	Four weeks	Pre-spray	Five weeks	Six weeks	Seven weeks	Eight weeks
**Mean ± S.E. of Amrasca biguttula biguttula /5 plants after different intervals post-spraying**										
**5%**	10.00 ± 0.58 a	4.00 ± 0.58 b	1.67 ± 0.88 b	1.33 ± 0.33 b	1.67 ± 0.33 b	1.67 ± 0.33 b	2.00 ± 1.15 b	1.67 ± 0.88 b	1.67 ± 0.88 b	1.67 ± 0.88 b
**2.5%**	10.00 ± 1.00 a	3.67 ± 1.20 b	2.33 ± 0.88 b	3.00 ± 0.58 b	4.00 ± 1.15 b	4.00 ± 1.15 b	3.67 ± 1.45 b	2.67 ± 0.88 b	2.33 ± 0.67 b	2.33 ± 0.67 b
**1.25%**	9.67 ± 0.33 a	3.67 ± 0.33 b	2.33 ± 0.33 b	2.00 ± 1.15 b	2.67 ± 0.88 b	2.67 ± 0.88 b	2.00 ± 1.00 b	2.67 ± 0.67 b	1.67 ± 0.67 b	1.67 ± 0.67 b
**control**	9.00 ± 0.58 a	7.67 ± 0.67 a	6.67 ± 0.33 a	10.67 ± 0.88 a	8.33 ± 0.33 a	8.33 ± 0.33 a	16.33 ± 0.88 a	12.33 ± 0.88 a	12.33 ± 0.33 a	10.67 ± 0.88 a
**F**	0.50	6.52	11.90	29.38	14.79	14.79	36.82	36.32	61.58	31.68
**Sig.**	0.69	0.02	0.00	0.00	0.00	0.00	0.00	0.00	0.00	0.00
**Mean ± S.E. of** ***Liriomiza trifolii*** **/5 plants after different intervals post-spraying**										
**5%**	22.00 ± 1.15 a	18.33 ± 1.86 ab	15.33 ± 1.33 b	11.00 ± 1.73 b	11.33 ± 3.38 b	11.33 ± 3.38 b	7.67 ± 0.88 c	6.00 ± 0.58 b	5.33 ± 0.33 b	4.33 ± 0.33 b
**2.5%**	22.67 ± 1.86 a	19.00 ± 2.52 ab	16.67 ± 2.40 ab	13.33 ± 2.19 b	13.67 ± 0.88 b	13.67 ± 0.88 b	13.67 ± 2.03 b	3.00 ± 1.73 b	2.33 ± 1.45 c	2.00 ± 1.53 b
**1.25%**	21.00 ± 1.53 a	15.67 ± 0.33 b	13.67 ± 0.33 b	10.33 ± 0.88 b	14.00 ± 1.73 b	14.00 ± 1.73 b	12.67 ± 0.88 a	3.00 ± 0.58 b	2.33 ± 0.33 c	1.67 ± 0.33 b
**control**	23.33 ± 0.88 a	23.33 ± 0.88 a	21.67 ± 1.45 a	22.33 ± 0.33 a	21.33 ± 1.20 a	21.33 ± 1.20 a	21.67 ± 1.33 a	22.00 ± 1.15 a	20.33 ± 0.88 a	21.67 ± 0.88 a
**F**	0.50	3.79	4.86	14.17	4.50	4.50	18.04	66.40	95.46	109.99
**Sig.**	0.69	0.06	0.03	0.00	0.04	0.04	0.00	0.00	0.00	0.00
**Mean ± S.E. of** ***Tuta absoluta*** **/5 plants after different intervals post-spraying**										
**5%**	8.67 ± 0.33 a	3.33 ± 0.33 b	3.67 ± 0.33 a	2.33 ± 0.33 b	1.33 ± 0.67 b	1.33 ± 0.67 b	1.67 ± 0.67 b	1.67 ± 0.88 b	1.33 ± 0.67 b	1.33 ± 0.67 b
**2.5%**	7.67 ± 0.33 ab	4.67 ± 1.86 ab	3.67 ± 1.86 a	4.00 ± 2.00 b	1.67 ± 0.88 ab	1.67 ± 0.88 ab	2.20 ± 1.00 b	0.33 ± 0.33 b	0.33 ± 0.33 b	0.33 ± 0.33 b
**1.25%**	8.67 ± 0.33 a	5.33 ± 2.03 ab	4.33 ± 2.33 a	3.33 ± 0.67 b	2.00 ± 1.15 ab	2.00 ± 1.15 ab	2.67 ± 1.45 b	1.00 ± 0.58 b	1.00 ± 0.58 b	1.33 ± 0.88 b
**control**	7.33 ± 0.33 b	8.67 ± 0.33 a	6.67 ± 0.33 a	7.67 ± 0.33 a	4.33 ± 0.33 a	4.33 ± 0.33 a	7.67 ± 0.33 a	7.33 ± 0.33 a	9.67 ± 1.45 a	7.33 ± 1.33a
**F**	4.25	2.65	0.89	4.64	2.78	2.78	6.87	30.97	25.91	13.18
**Sig.**	0.05	0.12	0.49	0.04	0.11	0.11	0.01	0.00	0.00	0.00

* Means followed by the same letter(s) within the same column are not significantly different at the 0.05 probability level.

**Table 4:** Effect of the *T. foenum-graecum* straw extract treatments against *Amrasca biguttula biguttula*,* Liriomiza trifolii*, and *Tuta absoluta* infesting tomato plants (first and second spray).

1st spray						2nd spray				
Concentrations	Pre-spray	One week	Two weeks	Three weeks	Four weeks	Pre-spray	Five weeks	Six weeks	Seven weeks	Eight weeks
**Mean ± S.E. of Amrasca biguttula biguttula /5 plants after different intervals post-spraying**										
**5%**	11.33 ± 0.33 a	4.67 ± 0.33 b	2.67 ± 0.33 b	0.67 ± 0.33 b	2.67 ± 0.67 b	2.67 ± 0.67 b	0.67 ± 0.67 b	0.67 ± 0.67 b	0.67 ± 0.67 b	0.67 ± 0.67 b
**2.5%**	10.67 ± 0.67 a	4.00 ± 1.15 b	2.00 ± 1.15 b	1.67 ± 0.88 b	3.67 ± 0.88 b	3.67 ± 0.88 b	2.00 ± 0.58 b	2.00 ± 0.58 b	2.00 ± 0.58 b	2.00 ± 0.58 b
**1.25%**	9.00 ± 0.00 b	3.33 ± 1.20 b	1.67 ± 0.88 b	2.33 ± 0.33 b	4.00 ± 1.15 b	4.00 ± 1.15 b	2.67 ± 0.88 b	2.67 ± 0.88 b	2.00 ± 0.58 b	2.00 ± 0.58 b
**control**	11.67 ± 0.33 a	10.67 ± 0.33 a	10.00 ± 0.58 a	9.67 ± 0.67 a	8.67 ± 0.33 a	8.67 ± 0.33 a	9.67 ± 0.33 a	10.00 ± 1.00 a	9.33 ± 0.33 a	10.33 ± 0.33 a
**F**	5.62	15.21	24.62	46.39	10.71	10.71	39.00	27.54	50.79	64.33
**Sig.**	0.03	0.00	0.00	0.00	0.00	0.00	0.00	0.00	0.00	0.00
**Mean ± S.E. of** ***Liriomiza trifolii*** **/5 plants after different intervals post-spraying**										
**5%**	21.33 ± 0.33 ab	11.33 ± 0.58 b	11.00 ± 0.58 b	10.33 ± 0.33 c	13.67 ± 1.20 ab	13.67 ± 1.20 ab	7.33 ± 0.33 c	6.00 ± 0.58 c	4.00 ± 0.58 c	2.33 ± 0.33 c
**2.5%**	20.33 ± 0.88 b	12.00 ± 1.73 b	9.67 ± 0.88 b	11.67 ± 0.67 c	8.67 ± 0.88 b	8.67 ± 0.88 b	8.00 ± 0.67 c	6.67 ± 0.67 c	4.67 ± 0.67 c	3.67 ± 0.67 c
**1.25%**	21.33 ± 0.33 ab	13.00 ± 1.15 b	11.00 ± 1.15 b	16.67 ± 1.20 b	14.67 ± 2.85 a	14.67 ± 2.85a	13.33 ± 1.45 b	11.67 ± 1.20 b	10.00 ± 1.15 b	8.00 ± 1.15 b
**control**	22.67 ± 0.33 a	20.67 ± 0.88 a	19.33 ± 0.33 a	22.33 ± 0.33 a	18.33 ± 0.88 a	18.33 ± 0.88 a	17.67 ± 0.67 a	18.33 ± 0.88 a	17.33 ± 0.33 a	17.33 ± 0.33 a
**F**	3.30	14.38	30.77	56.33	5.72	5.72	28.51	43.36	68.53	91.93
**Sig.**	0.08	0.00	0.00	0.00	0.02	0.02	0.00	0.00	0.00	0.00
**Mean ± S.E. of** ***Tuta absoluta*** **/5 plants after different intervals post-spraying**										
**5%**	7.67 ± 0.67 a	3.00 ± 0.58 b	1.33 ± 0.67 b	1.67 ± 0.33 c	2.00 ± 0.58 b	2.00 ± 0.58 b	1.00 ± 0.58 b	1.00 ± 0.58 b	1.00 ± 0.58 b	1.67 ± 0.67 b
**2.5%**	7.33 ± 0.88 a	3.33 ± 0.67 b	1.67 ± 0.88 b	3.67 ± 0.33 b	3.33 ± 0.67 b	3.33 ± 0.67 b	1.33 ± 0.88 b	1.00 ± 1.00 b	0.67 ± 0.67 b	2.67 ± 0.67 b
**1.25%**	7.67 ± 0.88 a	4.33 ± 0.33 b	3.33 ± 1.45 b	4.33 ± 0.88 ab	3.67 ± 0.33 b	3.67 ± 0.33 b	3.33 ± 1.86 b	3.67 ± 1.20 b	2.33 ± 0.88 b	3.33 ± 0.88 b
**control**	8.00 ± 1.00 a	7.33 ± 0.88 a	7.33 ± 0.33 a	8.33 ± 0.33 a	6.33 ± 0.33 a	6.33 ± 0.33 a	8.33 ± 0.33 a	8.33 ± 0.33 a	7.00 ± 0.58 a	7.33 ± 0.67 a
**F**	0.10	9.33	8.81	28.13	13.19	13.19	22.89	16.56	18.10	17.10
**Sig.**	0.96	0.01	0.01	0.00	0.00	0.00	0.00	0.00	0.00	0.00

* Means followed with the same letter(s) within the same column are not significantly different at the 0.05 probability level.

### Molecular Docking evaluation

**Fig. 4:**
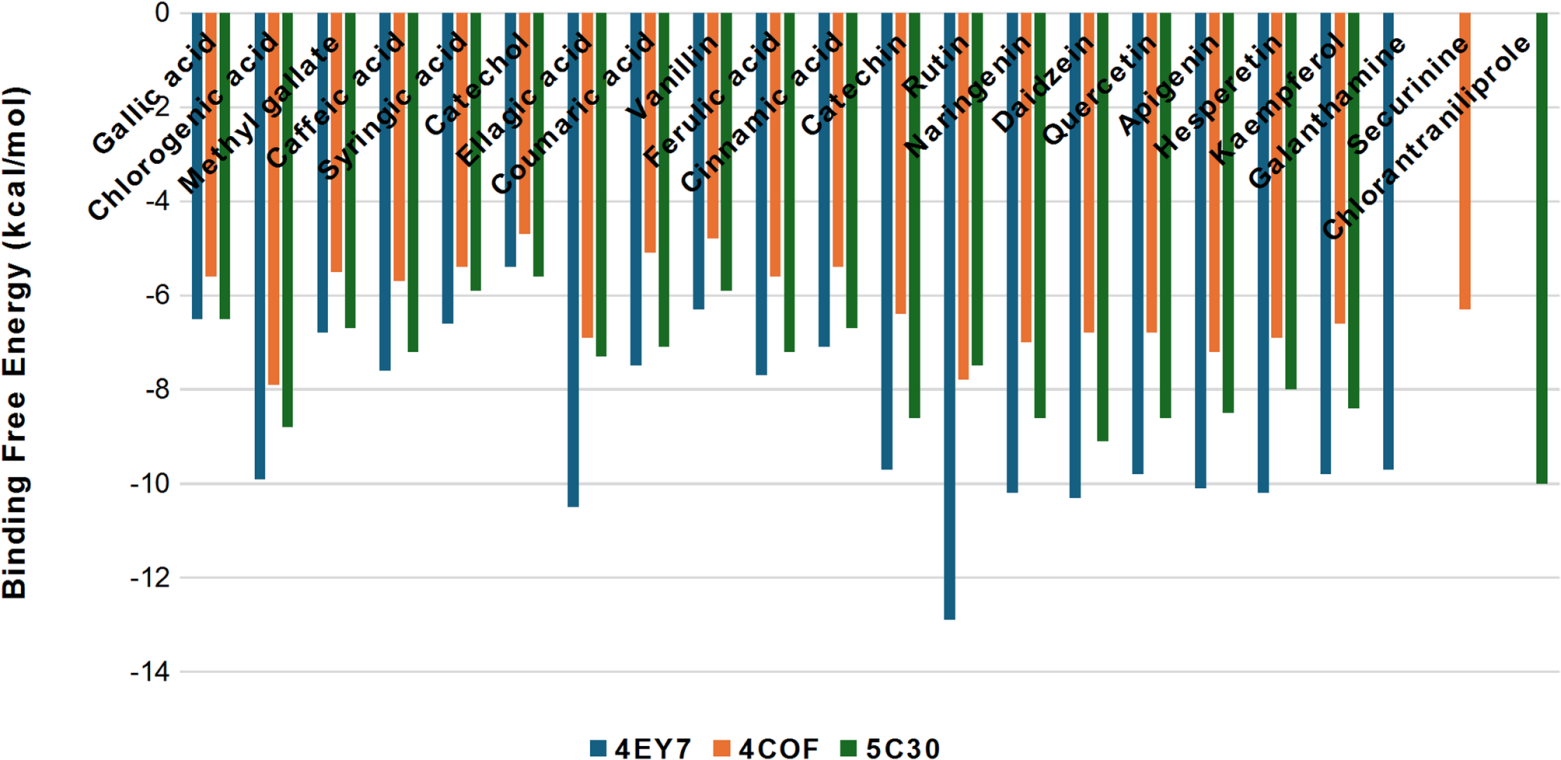
Docking scores for the interaction between phenols and flavonoids of *N. sativa* and *T. foenum-graecum* extracts and AChE (PDB: 4EY7), GABA_(A)_ (PDB: 4COF), and RyR (PDB: 5C30).

Molecular docking is used in the current study to predict the interaction between phenolic acids and flavonoids of *N. sativa* and *T. foenum-graecum* extracts and target enzymes: AChE (PDB ID: 4EY7), GABA_(A)_R-β3 (PDB ID: 4COF), and RyR (PDB ID: 5C30), based on the survey published by Rhaimi et al.^21^. The docking scores are presented in Fig. 4, where lower binding free energies indicate a higher affinity between the ligand and the receptor. Inhibition of AChE at synapses in arthropods reduces mobility and halts reproduction. Among the phenolic acid constituents of the extracts, the current study findings identified chlorogenic and ellagic acids (-9.9 and − 10.5 kcal/mol) as potent AChE inhibitors. At the same time, all the flavonoids were more powerful, especially rutin (-12.9 kcal/mol), compared to the control (Galanthamine) with − 9.7 kcal/mol (Fig. 4). Insecticides can bind to the ionotropic GABA receptor, preventing its interaction with glutamate-dependent chloride channels (GluCls) and disrupting arthropod synaptic transmission. Our analysis revealed that chlorogenic acid and rutin exhibit strong binding to the GABA receptor, with binding energies of -7.9 and − 7.8 kcal/mol, respectively, compared to the control (Securinine), which has a binding energy of -6.3 kcal/mol (Fig. 4). The RyR, a calcium release channel in insects, is vital for muscle contraction. Targeting it may lead to the development of new herbicides by causing paralysis in pests. Chlorantraniliprole, used as a control, showed the highest docking score with − 10.0 kcal/mol against RyR (PDB ID: 5C30). Still, both chlorogenic acid and daidzein have a comparable affinity to the same enzyme (-8.8 and 9.1 kcal/mol), suggesting they could yield similar beneficial effects (Fig. 4).

In agreement with our findings, Farouk et al.^16^ demonstrated that rutin, chlorogenic, and ellagic acids are among the most potent compounds against AChE, with a PDB ID of 1QON. The major constituents of the extract found in the phytochemical analysis of the hydroalcoholic extract of *Hypericum origanifolium* Willd (Hypericaceae) were hyperforin, hypericin, rutin, and chlorogenic acid, which the authors considered as possible active principles against GABA_A_^44^, in line with the results of the current study. To our knowledge, no published data concerning the molecular docking of phenolics and GABA(A)R-β3 or RyR has been found. Rhaimi et al.^21^ showed higher docking scores for sesquiterpenes compared to monoterpenes during the study of the insecticidal activity of *Salvia officinalis* essential oil. Phytocompounds from common weeds, such as *P. hysterophorus*, *L. camara*, and *A. sessilis*, were used as ligands against AChE Proteins of Aphids and Beetles. Docking results showed that kaempferol exhibited the least binding energy with the proteins of *R. padi*, *A. pisum*, and *M. persicae*^45^. Molecular docking simulations of *Euphorbia paralias* phytochemicals revealed the strong binding affinities of seven important flavonoids to key microbial and insecticidal target proteins of *Aphis gossypii* and *Amrasca biguttula*^46^. The high degree of concordance between computational predictions and experimental bioactivity results reinforces the therapeutic potential of these natural products.

**Fig. 5:**
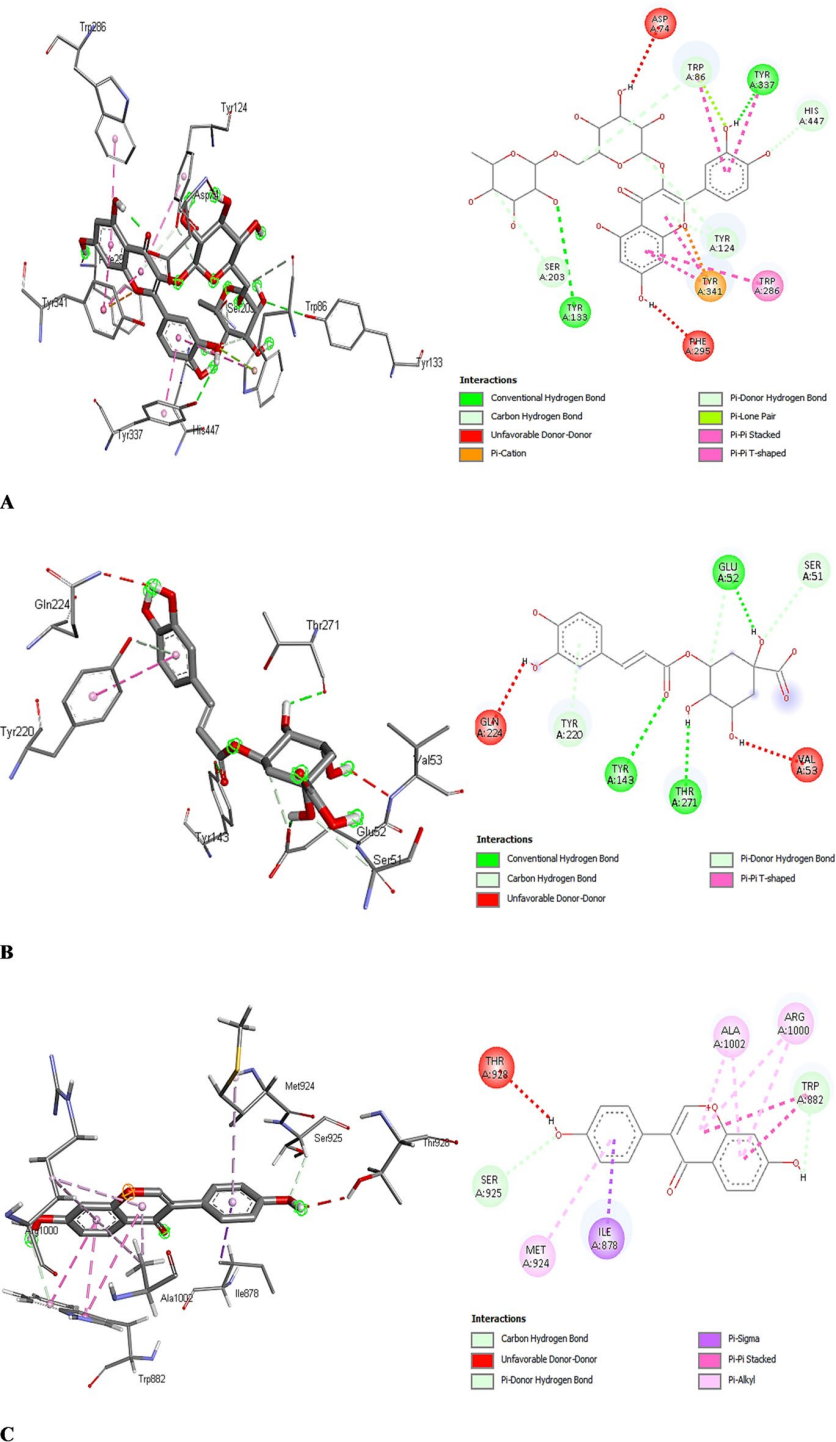
Docking profiles of (**A**) 4EY7-Rutin, (**B**) 4COF-Chlorogenic acid, and (**C**) 5C30-Daidzein complexes.

Figure 5 revealed the types of binding interactions of 4EY7-rutin (A), 4COF-chlorogenic acid (B), and 5C30-daidzein complexes, which showed the highest docking scores among all phytochemicals examined. Three conventional hydrogen bonds between the hydroxyl groups of TYR A:133, TYR A:143, and the ligand of 4EY7-rutin and 4COF-chlorogenic acid complexes, as proton donors, and hydroxyl, carbonyl, and carboxylate groups of the ligand, TYR A:337, THR A:271, and GLU A:52, as proton acceptors, were responsible for such a higher docking score (Figs. 5A and B). Charged O–H⋯O interactions, mainly between alcohols and carboxylic acids, were three times more common than neutral ones, with ligands more often acting as donors. Despite the polar -OH group, Tyr residues are quite hydrophobic. The -OH group of Tyr can form two hydrogen bonds, where it is a hydrogen bond donor twice as often as a hydrogen bond acceptor. In line with the interaction modes of Fig. 5A and B, the Tyr-OH group generally forms only a single intramolecular hydrogen bond, and the partner is most often a main-chain carbonyl or a side-chain carboxyl group. According to Pace et al.^47^, hydrogen bonds by tyrosine-OH groups contribute to protein stability.

Carbon-hydrogen bonds could be observed from the methylene and methine groups of HIS A:447, rutin, chlorogenic acid, SER A:51, and SER A:925 to the hydroxyl, carbonyl, and carboxylate groups of rutin, TYR A:124, TRP A:86, SER A:203, chlorogenic acid, THR A:271, GLU A:52, and daidzein (Figures A, B, and C). The interaction between carbon and hydrogen atoms, particularly C-H.O hydrogen bonds, can significantly determine binding affinity and stability in molecular docking. These interactions, where a hydrogen atom bonded to a carbon atom interacts with an electronegative atom, such as oxygen or nitrogen, are weak but important intermolecular forces^48^.

In line with TYR A:124, TYR A:220, and daidzein, which play the proton-donor role with rutin, chlorogenic acid, and daidzein pi-orbitals as acceptors, weak hydrogen bonds often contribute to protein-ligand binding subtly directionally, where the aromatic rings can act as acceptors of hydrogen bonds^49^. According to Brandl et al.^50^, the methyl groups of ILE are among the most prominent donors of the acceptors, as in the case of C^α^-H…Aro-π-interactions, as shown from ILE A:878 to the π-orbitals of daidzein (Fig. 5C).

Aryl rings are crucial for hydrophobic interactions in proteins, especially with amino acids like Tyr and Trp, which display their aromatic side chains at binding sites. Their shape and electronic properties facilitate favorable interactions, notably T-shaped edge-to-face and parallel-displaced stacking^49^. Hydrophobic π-π stacked and T-shaped interactions were observed between TRP A:286, TYR A:337, TYR A:341, TRP A:882, TRP A:86, TYR A:124, and TYR A:220 and the pi-orbitals of rutin, chlorogenic acid, and daidzein (Figs. 5A, B, and C). Finally, hydrophobic π-alkyl interactions could be observed between the π-orbitals of daidzein and the alkyl groups of ARG A:1000, ALA A:1002, and MET A:924 (Fig. 5C).

## Conclusion

Agricultural waste poses significant environmental and health challenges. This study shows that flavonoid- and phenol-rich extracts have a promising insecticidal effect under greenhouse conditions. Significant differences in pest infestations were observed between the treatment groups and control after sprays, with a marked reduction in pests by the third week post-treatment. Notable reductions in *Amrasca biguttula biguttula*, *Liriomyza trifolii*, and *Tuta absoluta* were found with *N. sativa* and *Trigonella foenum-graecum* straw extracts after four weeks. Key phenolic acids (ellagic, chlorogenic, gallic) and flavonoids (naringenin, rutin, catechin) likely contributed to this insecticidal activity. In-silico studies confirmed that these compounds demonstrated favorable docking scores against key enzymes. This research supports the use of agricultural waste as eco-friendly bioinsecticides, potentially alleviating issues related to waste accumulation. In addition, it opens up new perspectives on the applications of such extracts in the protection of other crops. Meanwhile, further enzymatic investigations could be helpful and necessary to relate and interpret in-silico and the enzyme activity. Furthermore, future studies should evaluate the effects of botanical extracts on non-targets, including microorganisms, arthropods, and enzymes, as well as their biodegradation mechanisms.

## Data Availability

Data is provided within the manuscript.
